# Effects of Reappraisal and Self-Compassion Expressive Writing on Emotion Regulation

**DOI:** 10.1177/17470218251392569

**Published:** 2025-10-18

**Authors:** Teresa Jacques, Rui A. Alves

**Affiliations:** 1 Faculty of Psychology and Education Sciences, Center for Psychology at the University of Porto, Portugal

**Keywords:** expressive writing, emotion regulation, writing instructions, autonomous nervous system, heart-rate variability

## Abstract

Expressive writing is the disclosure of negative events in a safe and non-threatening environment while focusing on the feelings and emotions associated with an experience. Studies have proposed that alternative expressive writing instructions can influence expressive writing outcomes and shed light on the benefits of the intervention. Thus, we created two novel expressive writing instructions susceptible to inducing emotion regulation: a reappraisal and a self-compassion instruction. Sixty-six college students at a university were randomly assigned to either reappraisal or a self-compassion expressive group. Positive and negative affect, emotion regulation, anxiety, and alexithymia were measured before and after writing. Electrocardiogram was recorded during the experiment to examine the effects of the writing exercise on the Autonomous Nervous System. We found that expressive writing decreased Heart-Rate (HR) (*d* = 0.63) and alexithymia (*d* = 0.55) in both groups. In addition, the LF/HF ratio was higher in the reappraisal expressive group (*d* = 0.70). These findings support the use of expressive writing as a tool to promote emotion regulation.

## Introduction

Does writing about negative life events improve certain aspects of mental health? Pennebaker and Beall (1986) created the expressive writing (EW) paradigm to answer this question. They considered that discussing negative events with others was difficult, which led to the inhibition of feelings and emotions. Thus, expressive writing gave people a safe way to disclose those events and emotions. Disclosing events is important since people must “work through” painful/traumatic episodes to heal from them (Mordechay et al., 2019).

Originally, the expressive writing paradigm meant writing about the most traumatic event of someone’s life, for 4 consecutive days, for 15 min each day (Pennebaker, 2018; Pennebaker & Chung, 2007). Throughout the years, researchers have consistently shown positive results of EW across different study designs and modifications of the original writing instruction (Frattaroli, 2006; Smyth, 1998; Sloan & Marx, 2018, Valtonen, 2020; see Lepore & Smyth, 2002). These modifications of the original paradigm came as an attempt to improve its effectiveness (Nazarian & Smyth, 2013), by promoting therapeutic processes (Danoff-Burg et al., 2010).

Research suggests that EW effects vary as a function of individual differences. Therefore, it is essential to understand the conditions under which expressive writing works and how to maximize its benefits (Lu & Stanton, 2010). EW instructions should also direct individuals to identify their emotions and enable the reflection of how the event changed their thoughts and beliefs, eventually leading to emotional habituation (Nazarian & Smyth, 2013). Instructing participants to focus on cognitive restructuring of stress or trauma has shown increases in insight (van Middendorp et al., 2007).

In this light, the present study proposes two alternative expressive writing prompts, both aiming to induce emotion regulation: (a) Reappraisal EW and (b) Self-Compassion EW. Cognitive reappraisal (Gross, 2002) can be defined as a positive change in the evaluation of stressors and/of the self (Lu & Stanton, 2010), and a possible pathway to reduce the impact of stress and produce beneficial effects through expressive writing (Lazarus & Folkman, 1984). Cognitive reappraisal is one of the five emotion regulation strategies of the process model of emotion regulation (Gross, 2002) and is defined as a type of cognitive strategy that helps to change the constructs related to an emotional situation (Gross, 2002). Reappraisal is a commonly used form of emotion regulation centering on people’s attempts to reframe how they are thinking about an emotional situation so that they can feel better (Ford et al., 2017). Interventions that promote reappraisal of adverse events are likely to help prevent future depressive episodes (Gortner et al., 2006). Reappraisal is also one of the most widely studied emotion-regulation strategies, and decades of research have shown reappraisals’ benefits for emotional, social, cognitive, and physiological outcomes (Aldao et al., 2010). Given this accumulated evidence, it is tempting to draw two conclusions about reappraisal: (a) that people can use it easily and (b) that people should use it frequently (Ford & Troy, 2019).

Another possible way to induce positive writing effects is self-compassion, closely related to both physical and mental health (Wong & Mak, 2016). Self-compassion is the act of accepting one’s own suffering, while being kind toward oneself (Neff, 2003). Self-compassion has three dimensions: (a) self-kindness; (b) common humanity – knowing that suffering is a common human experience and (c) mindfulness, with the acceptance of suffering while still being balanced. Self-compassion has been shown to lead to decreased negative emotions and increased positive emotions (Berking & Whitley, 2014). Self-compassion helps raise awareness of emotions and changes feelings of distress to kindness and understanding, helping transform negative emotions into more positive ones (Neff, 2003). Self-compassion has been linked to reduced mental health symptoms (Wilson et al., 2019). More specifically, higher levels of self-compassion were correlated with lower levels of depression, anxiety, and stress in adults (MacBeth & Gumley, 2012), overall psychological well-being (Zessin et al., 2015), life satisfaction, emotional intelligence, happiness, and lower suppression (Neff, 2009). Self-compassion has also been used as a treatment target for people who avoid thinking and living emotional experiences.

Measuring emotion regulation self-report measures before and after expressive writing can also be important. Expressive suppression, anxiety, and alexithymia are common self-report measures in EW studies. Expressive suppression is the inhibition of an ongoing emotion-expressive response (Gross, 2002). Suppressing negative feelings has been associated with increased negative emotion (Dalgleish et al., 2009) and it could mean unsuccessful cognitive processing (Lepore et al., 2002). Moreover, assessing whether anxiety and alexithymia can improve after expressive writing also seems particularly important. This is so since expressive writing has been shown to have positive effects on anxiety (Park et al., 2014; Shen et al., 2018) and on disrupted emotional awareness or alexithymia (Baikie, 2008; Hogeveen & Grafman, 2021), in particular, it has been shown that higher alexithymia is associated with lower usage of cognitive reappraisal, approaching problems, and seeking social support (Preece et al., 2023).

Measuring heart activity using the electrocardiogram (ECG) is a prime method to study emotions; this is because the ECG reflects autonomous nervous system (ANS) activity (Selvaraj et al., 2013) by using the dynamics of the sympathetic (SNS) and parasympathetic (PNS; vagal) branches of the ANS. Heart rate has been shown to drop to levels below baseline following expressive writing (Pennebaker et al., 1997) and to increase after expressive writing (Sloan et al., 2007). Meanwhile, heart-rate variability (HRV) describes changes in HR (Fooken, 2017), giving information on the interplay between the SNS and the PNS (Thayer et al., 2012). The SNS is responsible for fight-or-flight responses, becoming dominant during psychological stress, by producing physiological arousal to help cope with the task at hand (Appelhans & Luecken, 2006; Fooken, 2017). The PNS is responsible for rest and relaxation (Fooken, 2017), being more active during periods of perceived safety (Appelhans & Luecken, 2006). HRV is therefore indicative of the capacity for regulated emotional responding (Appelhans & Luecken, 2006; Thayer et al., 2012). HRV can be measured using: (a) time-domain and (b) frequency-domain measures: (a) Time-domain measures include the standard deviation of normal-to-normal intervals (SDNN; measured in ms), representing overall autonomic activity (Pichot et al., 2016). SDNN has been shown to remain the same (Jacques et al., 2020; Jacques & Alves, 2024) and to increase as a result of expressive writing, after participants were primed to believe they would not get into graduate school (Seeley et al., 2016). Another measure, the square root of the mean squared differences of successive N–N intervals (RMSSD; measured in ms; Malik et al., 1996), represents PNS activity (Malik et al., 1996). RMSSD increases are related to the use of emotion reappraisal in non-expressive writing studies, (Knepp et al., 2015; Shahane et al., 2020), and in expressive writing, it has been shown to remain the same (Jacques & Alves, 2024; Jacques et al, 2020). (b) Frequency-domain measures are low frequency (LF; frequency range: 0.04–0.15 Hz); high frequency (HF; frequency range: 0.15–0.4 Hz; PNS activation; ms^2^; Malik et al., 1996) power and LF/HF ratio. LF/HF ratio is a measure of sympathovagal balance (Malliani, 1999; Malik et al., 1996), with higher values representing sympathetic predominance (Pichot et al., 2016). RSA (a synonym of HF) has been shown to increase after traditional expressive writing (Bourassa et al., 2017; Sheerin et al., 2018). Finally, LF/HF ratio has been shown to increase during and after traditional expressive writing (Jacques & Alves, 2024; Jacques et. al., 2020), which seems indicative of emotion regulation. Outside of expressive writing, an increased LF/HF ratio has been linked to positive psychotherapy outcomes (Steffen et al., 2021), higher emotional control (Kim et al., 2019), higher well-being (Shiga et al., 2022), and improved emotion regulation (Peng et al., 2019). Studying emotion together with psychophysiology has become increasingly important, and as far as we know, no other study has measured psychophysiology during reappraisal and/or self-compassion expressive writing.

Even though it is known that expressive writing has benefits, how these benefits occur is not yet fully understood. Comparing two novel expressive writing prompts that specifically induce the use of emotion regulation strategies is an important step to understanding if these benefits are due to emotion regulation. In addition, measuring psychophysiological responses can help better understand the physical impact of the tasks on emotion regulation. We also wanted to study the impact of the two writing prompts on anxiety symptoms and alexithymia characteristics. Participants were randomly assigned to two groups, writing about either a traumatic event while using reappraisal or self-compassion. We expected participants in both groups to show significantly higher negative affect and lower positive affect following the expressive writing task (manipulation check), since this is the typical finding in expressive writing studies (see Baikie & Wilhelm, 2005; Pennebaker & Beall, 1986; Smyth, 1998).

We expected suppression to decrease and reappraisal to increase in both expressive groups and reappraisal to be higher in the reappraisal group (H1). We expected both anxiety and alexithymia to be lower in both groups when compared to baseline (H2).

For the psychophysiological measures, we expected participants in both groups to show increased SNS activation, due to the heavy emotional load of writing about a traumatic or stressful life event (H3a). We also expected the reappraisal group to show lower PNS activation (RMSSD; HF) and higher LF/HF ratio than the self-compassion group – since the LF/HF ratio is thought to index emotion regulation (Porges et al., 1994; H3b).

## Method

### Participants

Sixty-six college undergraduates were recruited as decided a priori. Estimates of sample size were deemed appropriate a priori by G*Power 3.1 (Faul et al., 2009), as a sample size of 66 participants was estimated to achieve 0.85 power (*f* = 0.30). Previous reviews and meta-analysis have shown both average (Smyth’s, 1998) and small (Frattaroli’s, 2006) effect sizes. Participants were randomly assigned to one of two groups: an expressive group writing using reappraisal (*n* = 32; range = 18–42 years, *M* = 20.04, *SD* = 4.74; 12.5% male), an expressive group writing using self-compassion (*n* = 34; range = 18–31 years, *M* = 19.15, *SD* = 2.44; 0% male). Participants were treated ethically and received course credits for their participation. Ethical clearance was obtained from the university’s ethics committee (2021/07-08b). All participants were bachelor students at the same university, resided in the same district, and all were native Portuguese speakers.

### Measures

#### Positive and Negative Affect Schedule

The positive and negative affect schedule (PANAS; Galinha & Pais-Ribeiro, 2005; Watson et al., 1988) was used before and after writing. PANAS is divided into two scales: positive and a negative mood scale, with 10 items each. Each item is answered using a 5-point Likert scale (min = 10; max = 50).

#### Emotion Regulation Questionnaire, General Anxiety Disorder-7, Toronto Alexithymia Scale-20

The emotion regulation questionnaire (ERQ; Gross & John, 2003; Brandão et al., 2017) measured emotion regulation (expressive suppression, 4 items; min = 4; max = 28; and cognitive reappraisal, 6 items; min = 6; max = 42), answered using a 7-point Likert scale. The Generalized Anxiety Disorder Scale (GAD-7; Sousa et al., 2015; Spitzer et al., 2006) measured generalized anxiety before writing and 1 month after writing. This is a Likert scale questionnaire (min = 0; max = 21). The Toronto Alexithymia Scale-20 (TAS-20; Bagby et al., 1994; Praceres et al., 2000) measured alexithymia before writing and 1 month after writing. The TAS-20 is a 20-item scale, using a 5-point Likert scale (from 1 to 5; min = 20; max = 60).

#### ECG Recording

Raw ECG was collected using the ECG Bionomadix transmitter, together with the Bionomadix logger (Biopac Systems, CA; sampling rate 1,000 Hz; bandpass filter 0.5–35 Hz AcqKnowledge 5.0). The data was analyzed in HRVanalysis 1.2 (Pichot et al., 2016), using wavelet transform through local linear analysis, by dividing the recording into five parts: the last 5 min of baseline, 15 min of writing divided into three parts (averaged to one writing moment), and 5 min of post-writing. The following measures were extracted: HR; RMSSD; RSA(HF); SDNN, and LF/HF ratio.

### Writing Prompts

For the reappraisal group, we asked participants to write about the positive and negative consequences of the experience, since cognitive reappraisal involves reframing an emotional stimulus to change its emotional impact (Gross, 2015). In addition, previous literature suggests that reappraisal is an effective way to change both negative and positive emotions over the short and longer term (Troy et al., 2018), and therefore, this positive/negative emotion dichotomy is necessary for a cognitive reappraisal prompt. For the self-compassion group, we asked participants to write about their experience with understanding, acceptance, and compassion for themselves (for full instructions, see https://osf.io/ka58m/overview). As far as we are aware, no other study has developed self-compassion expressive writing instructions before.

### Procedure

The study had three moments: (a) an initial online questionnaire, (b) an in-person session, when writing was done, and (c) an online follow-up questionnaire 1 month later. Participants were recruited in undergraduate-level courses via email. In moment 1, participants answered an initial questionnaire that included the ERQ, GAD-7, and TAS-20. In moment 2, participants began by answering the PANAS before and after the task. ECG was collected before, throughout, and after writing in an enclosed booth to secure privacy and confidentiality. ECG recording was done by placing three electrodes according to a lead III configuration (see Berntson et al., 2007, p. 184; one electrode on the inside of the bottom of each leg and one electrode in the non-dominant hand). Before instructions were delivered, participants were asked to sit and rest for 10 min (baseline). Instructions were given in an envelope to ensure the experimenter was blind to each participant’s experimental condition (double-blinding). Participants were randomly assigned to the two groups, with the second author, who did not participate in any moment of the data collection, shuffling the envelopes to ensure unbiased randomization. Both groups were asked to write for 15 min. After writing, they stayed seated and at rest for 5 min and asked to complete PANAS and the ERQ. Every participant was debriefed after participating. In the one-month follow-up, participants answered the moment (c) follow-up questionnaire, which included the ERQ, GAD-7, and TAS-20.

### Data Analysis

The groups were compared on PANAS, ERQ, GAD, TAS-20, HR, RMSSD, SDNN, HF, and LF/HF ratio. Outliers were identified through box plot analysis and removed from the database, as decided a priori. A repeated measures Analysis of Variance (ANOVA) was conducted on PANAS, with positive and negative affect as a repeated measures factor, separately, and topic (reappraisal, self-compassion) as a between-subjects factor. The ERQ was analyzed using a repeated measures ANOVA with before writing (Moment 1) as covariate, moments (Immediately after writing – Moment 2; 1 month after writing/follow-up – Moment 3) as within-subjects factor, and topic (reappraisal, self-compassion) as between-subjects factor. The GAD-7 and TAS-20 were analyzed using repeated measures ANOVA with moments (Before writing – Moment 1; 1 month after writing/follow-up – Moment 3) as a within-subjects factor and topic (reappraisal, self-compassion) as a between-subjects factor. HR, RMSSD, SDNN, HF, and LF/HF ratio were analyzed independently using a repeated measures ANOVA, with the time period (writing, post-writing) as a within-subjects factor, topic (reappraisal, self-compassion) as a between-subjects factor, and baseline as a covariate. Statistical analyses were conducted with the α threshold of .05. Raw data and outlier exclusions are available on the Open Science Framework: https://osf.io/ka58m/overview.

## Results

### Descriptive Statistics

For the manipulation check, both groups had equivalent positive and negative affect before writing. For LF/HF ratio, the lowest value was 1.77 in the reappraisal group before writing and the highest was also in the reappraisal group, after writing (2.71). For Alexythemia the lowest value was in the self-compassion group after writing (53.44) and the highest in the same group before writing (56.21). For full descriptive statistics see Table 1.

**Table 1.** Descriptive Statistics for the Entire Sample Divided by Experimental Groups.

Measures	Reappraisal	Self-compassion
	*M(SD)*	*M(SD)*
M1_Positive Affect	27.00 (5.83)	27.79 (7.25)
M2_Positive Affect	22.16 (7.63)	23.79 (9.22)
M1_Negative Affect	12.34 (2.19)	13.24 (3.28)
M2_Negative Affect	19.66 (9.26)	16.32 (4.77)
M1_Expressive Supression	15.00 (5.10)	14.15 (5.29)
M2_ExpressiveSupression	15.84 (5.18)	14.97 (6.19)
M3_ExpressiveSupression	14.52 (5.96)	15.16 (5.11)
M1_Cognitive Reappraisal	28.81 (6.35)	27.59 (7.16)
M2_Cognitive Reappraisal	28.97 (5.99)	27.74 (7.19)
M3_Cognitive Reappraisal	29.68 (2.48)	27.84 (7.90)
M1_GAD	8.47 (5.13)	7.59 (4.56)
M3_GAD	8.59 (4.17)	7.88 (4.51)
M1_TAS20	56.13 (11.49)	56.21 (10.92)
M3_TAS20	54.52 (9.26)	53.44 (10.10)
B_Mean HR (bpm)	86.21 (6.16)	87.29 (11.88)
W_Mean HR (bpm)	91.63 (8.45)	93.60 (9.73)
PW_Mean HR (bpm)	86.97 (6.29)	88.65 (12.73)
B_SDNN (ms)	51.44 (9.19)	59.27 (21.75)
W_SDNN (ms)	43.42 (12.53)	45.54 (19.10)
PW_SDNN (ms)	61.26 (12.83)	61.02 (22.90)
B_RMSSD (ms)	33.16 (7.45)	36.91 (12.34)
W_RMSSD (ms)	30.92 (8.09)	28.59 (7.20)
PW_RMSSD (ms)	33.56 (10.01)	33.01 (11.68)
B_HF (ms^2^)	426.24 (227.32)	577.09 (378.59)
W_HF (ms^2^)	291.67 (174.53)	336.19 (265.96)
PW_HF (ms^2^)	453.31 (237.42)	588.22 (414.91)
B_LF/HF (ms^2^/ms^2^)	1.77 (0.88)	1.80 (0.88)
W_LF/HF (ms^2^/ms^2^)	2.35 (0.90)	1.97 (0.77)
PW_LF/HF (ms^2^/ms^2^)	2.71 (1.40)	2.41 (1.06)

*Note.* HF = high frequency; LF = low frequency; GAD = Generalized anxiety questionnaire; TAS20 = Toronto Alexithymia Scale – 20; RMSSD = root mean square of the successive N–N interval differences; SDNN = *SD* of normal-to-normal (N–N) intervals; M1 = Before writing; M2 = immediately after writing; M3 = 1 month after writing; B = Baseline; W = Writing; PW = Post writing.

### Manipulation Check

The Repeated Measures ANOVA showed that both groups had lowered positive affect from before to after writing, with a significant effect of moments, *F*(1,64) = 24.99, *p* < .001, η²_*p*_ = .28, *d* = 1.25. There was no effect of moment × group or between-group effects (*F* < 1).

For negative affect, there was a significant effect of moments, *F(*1,64) = 37.06, *p* < .001, η²_*p*_ = .37, *d* = 1.53, and a moments x group interaction, *F*(1,64) = 6.116, *p* = 016, η²_*p*_ = .09, *d* = 0.63, with both groups increasing negative affect after writing.

### Emotion Regulation (H1), Anxiety and Alexithymia (H2)

The repeated measures Analysis of Covariance (ANCOVA) showed no effect for suppression, for moments *F*(1,58) = 3.65, *p* = .061, group, or moments x group interaction (*F* < 1). The same result was found for reappraisal, for moments, *F*(1,50) = 1.40, *p* = .243, group or moments x group interaction (*F* < 1). There was also no effect of moments, group, or moments group interaction for Anxiety (*F* < 1). There was a significant effect of moments for Alexithymia, *F*(1,58) = 4.26, *p* = .044, η²*p* = .068, *d* = 0.54 (a medium effect size; Cohen,1988).

### Effects of Writing on SNS and PNS Activation (H3a and H3b)

**Figure 1.**
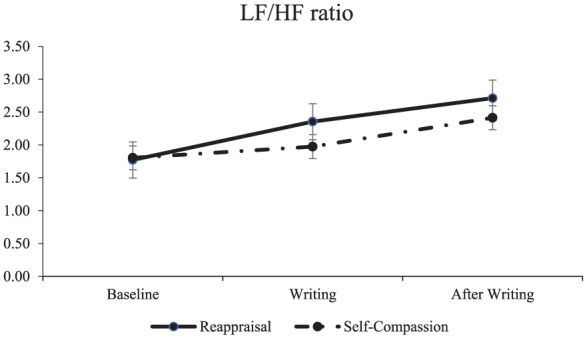
LF/HF ratio across moments for the reappraisal and the self-compassion group. *Note.* The Figure displays the LF/HF ratio, in ms^2^/ms^2^, for baseline, during expressive writing (Writing), and after writing, for both the reappraisal and the self-compassion group. Error bars represent standard error.

The repeated measures ANCOVA showed that HR was lower after writing for both groups, *F*(1,44) = 4.09, *p* = .049, η²_*p*_ = .09, *d* = 0.63 (a medium effect size; Cohen,1988). For SDNN, there was no interaction between moments x group moments *F*(1,44) = 1.31, *p* = .259, or effect of group (*F* < 1). There was no effect of moments or group (*F* < 1) or moments x group interaction, *F*(1,43) = 1.93, *p* = .172. (H1a). For RMSSD, there was no effect of moments, group, or moment x group interaction (*F* < 1). For HF there was no effect of moments, *F*(1,40) = 1.38, *p* = .247, group, *F*(1,40) = 1.04, *p* = .313, or moments x group interaction, (*F* < 1) (H1b). Finally, the LF/HF ratio was significantly different between groups, *F*(1,42) = 5.09, *p* = .029, η²_*p*_ = .108, *d* = 0.70 (a medium effect size; Cohen, 1988), with the reappraisal group having a higher LF/HF ratio by .496 ms^2^/ms^2^. There was no effect of moments *F*(1,42) = 1.54, *p* = .222, or moments x group interaction (*F* < 1) (H1c) (Figure 1).

## Discussion

With this study, we wanted to study the impact of two expressive writing prompts (reappraisal vs self-compassion) on emotional regulation, anxiety symptoms, and alexithymia characteristics. Reappraisal is an important emotion regulation strategy since it is one of the most widely studied emotion-regulation strategies, and it has shown benefits for emotional, social, cognitive, and physiological outcomes (Aldao et al., 2010).

Self-compassion could be equally important, since it has been shown to lead to decreased negative emotions and increased positive emotions (Berking & Whitley, 2014). In addition, self-compassion helps awareness of emotions and changes feelings of distress to kindness and understanding, helping transform negative emotions into more positive ones (Neff, 2003).

We found that HR decreased after writing for both groups and that the reappraisal group had a higher LF/HF ratio, an indicator of emotion regulation (Peng et al., 2019), in addition, both groups, showed lower alexithymia after writing.

### Manipulation Check

In this study, expressive writing led to a decrease in positive affect and an increase in negative affect after writing, for both groups. This replicates past EW studies (see Baikie & Wilhelm, 2005; Jacques et al., 2020; Konig et al., 2014; Pascual-Leone et al., 2016). This result can be explained since experiencing negative emotions to a certain optimal extent, not too much and not too little, is thought to be crucial to the process of coming to terms with an emotion-laden experience (Mordechay et al., 2019)

### Emotion Regulation (H1)

Our study showed no effects on suppression and reappraisal. Other studies found that reappraisal expressive writing interventions lead to reduced negative emotions and increased positive emotions in comparison to the control groups (K. Wang et al., 2021). Different from our study, self-report reappraisal was not measured, and effects were measured through response to COVID-19 related pictures and positive and negative emotions. These differences in measures included in the study design, might explain the lack of effects in our study. It has also been found that reappraisal increased, and suppression decreased 1 month after expressive writing (Cheung et al., 2021). In this study, participants completed traditional expressive writing, for 3 consecutive days, 20 min each day. In our study, we purposely changed writing instructions and participants only wrote once, for 15 min, which might explain differences in results.

### Anxiety and Alexithymia (H2)

Our study showed no effects on anxiety, contrary to previous studies (Park et al., 2014; Shen et al., 2018). In a previous study that used reappraisal expressive writing, compared to irrelevant writing, both groups showed decreased anxiety after writing (F. Wang et al., 2015); however, differently from our study, the participants were primed to be anxious before writing. Finally, we found that alexithymia decreased after writing for both groups. A previous study also found positive results of expressive writing on alexithymia (Baikie, 2008). Moreover, it has previously been shown that participants who completed expressive writing and had higher alexithymia before writing showed higher positive affect in comparison to those who did not (Páez et al., 1999). This positive outcome of lower alexithymia after both prompts was expected since the opposite has been shown before (higher alexithymia related to lower usage of cognitive reappraisal; Preece et al., 2023), and since self-compassion helps awareness of emotions and is thought to change negative thoughts into positive ones (Neff, 2003).

### Effects of Expressive Writing on SNS and PNS (H3a and H3b)

We found that HR decreased for both groups. A heart rate decrease after expressive writing has also been shown before (Pennebaker et al., 1997). SDNN and RMSSD have been shown to remain the same in previous studies (Jacques & Alves, 2024; Jacques et al., 2020). In another study, SDNN was shown to increase after expressive writing (Seeley et al., 2016). The differences might be because, in Seeley et al., participants had a negative prime before expressive writing, which we did not have in the current study. We also found that the LF/HF ratio was higher in the reappraisal group in comparison to the self-compassion group. LF/HF ratio has been shown to increase during and after traditional expressive writing in two previous studies (Jacques & Alves, 2024; Jacques et al., 2020). The LF/HF ratio has been linked to higher emotion regulation (Peng et al., 2019), well-being (Shiga et al., 2022), and positive psychotherapy outcomes (Steffen et al., 2021)

### Implications and Limitations

Our study has important implications for research and practice. First, we found that the reappraisal group had a higher LF/HF ratio in comparison to the self-compassion group. Since a higher LF/HF ratio is thought to index higher emotional control (Kim et al., 2019), higher well-being (Shiga et al., 2022), and improved emotion regulation (Peng et al., 2019), we suggest that reappraisal expressive writing is likely to induce emotion regulation. In addition, this result has been shown before in typical expressive writing (Jacques & Alves, 2024; Jacques et al., 2020). This has important implications for clinical practice since it shows that this prompt might be a good option when wanting to improve emotion regulation in patients. In addition, it shows that expressive writing decreased HR, and therefore SNS, in both prompts, suggesting that both prompts can be used to reduce arousal. Finally, our study shows that both prompts decreased alexithymia, showing that both prompts might be used when participants have difficulties identifying emotions.

These results should be viewed under the following limitations: The participants from this study were university students, which can limit generalization. Future studies should branch out outside of only one department or academia in general, moving to clinical populations in hospitals and the community. Second, adding a self-compassion questionnaire could be helpful to analyze the self-perceived effects of the task. Third, we did not study which words were used during moments of high psychophysiological arousal. Future studies could explore the linguistic contents produced, specifically, if a higher LF/HF ratio is associated with specific words/sentences. Fourth, the type of instructions used was different than previous expressive writing studies. Future studies should replicate the study to further support the results found. In addition, we did not include a specific measure of understanding of the writing instructions. Fifth, participants wrote about traumas that happened at different periods in their lives. This could have affected emotion regulation results, since some participants might have had time to regulate their emotions prior to participating in the study. Sixth, we do not know if participants were currently in psychotherapy. This should be controlled in future studies.

Finally, our study found an increased LF/HF ratio, which could suggest increased emotion regulation (Kim et al., 2019) during expressive writing, but other indexes, such as SDNN, RMSSD, and HF, did not yield significant differences between groups.

In conclusion, we conducted a study to observe if expressive writing instructions using reappraisal or self-compassion affected emotional regulation, anxiety symptoms, and alexithymia characteristics. We found that alexithymia was lower in both groups after writing. In addition, we found that HR was lower in both groups after writing, and the LF/HF ratio was higher in the reappraisal group, in comparison to the self-compassion group

These are promising results, as they suggest that both instructions could be used to improve alexithymia and that reappraisal-based expressive writing might be better suited to promote emotion regulation when there are difficulties identifying emotions, which is particularly important for clinicians interested in using expressive writing.
